# COVID-19 Mortality in Vaccinated vs. Unvaccinated Liver & Kidney Transplant Recipients: A Single-Center United States Propensity Score Matching Study on Historical Data

**DOI:** 10.3390/vaccines10111921

**Published:** 2022-11-13

**Authors:** Hailey Hardgrave, Allison Wells, Joseph Nigh, Garrett Klutts, Derek Krinock, Tamara Osborn, Sushma Bhusal, Mary K. Rude, Lyle Burdine, Emmanouil Giorgakis

**Affiliations:** 1 Department of Surgery, University of Arkansas for Medical Sciences, Little Rock, AR 72205, USA; 2 Division of Nephrology, UAMS, Little Rock, AR 72205, USA; 3 Division of Hepatology, UAMS, Little Rock, AR 72205, USA; 4 Division of Solid Organ Transplantation, UAMS, Little Rock, AR 72205, USA

**Keywords:** COVID-19, vaccination, solid organ transplant, COVID-19 mortality, propensity score matching

## Abstract

Introduction: Existing studies report variable impact of vaccination on Coronavirus Disease (COVID-19) morbidity and mortality in solid organ transplant (SOT) recipients. This study aimed to perform a propensity score matching (PSM) analysis on COVID-19 survival of vaccinated and unvaccinated SOT patients who contracted the disease at a single US academic transplant center. Methods: All consecutive COVID-19 positive cases on adult liver, kidney or combined liver-kidney recipients were identified and demographics, comorbidities, immunosuppression, COVID-19 treatment and hospitalization status, COVID-19 vaccination status, and early mortality recorded. PSM study was performed on age and sex for completed vaccination status at time of infection, followed by multivariable analysis and survival curve plotting. Results: 144 SOT patients were diagnosed with COVID-19, with 98 unvaccinated. PSM reduced study number to 101. Matched data multivariable analysis for 60-day mortality identified age and post-kidney transplant status to significantly increase 60-day mortality odds (OR 1.22, *p* < 0.001 and OR 40.93, *p* < 0.001, respectively). Kaplan–Meier analysis showed inferior post-infection survival in the unvaccinated group [(30 days; vaccinated vs. unvaccinated 97.8% vs. 89.1%, respectively; *p* = 0.089) (60 days; 97.8% vs. 83.6%, respectively; *p* = 0.019)]. Conclusions: Matched data survival analysis demonstrated inferior survival in the unvaccinated group, supporting COVID-19 vaccination in SOT recipients.

## 1. Introduction

Since the roll out of the first Coronavirus Disease 2019 (COVID-19) vaccines in late 2020, there has been hope for decreased COVID-19 morbidity and mortality across the world. While this hope has become reality for the majority, specific populations like solid organ transplant (SOT) recipients may not be afforded the same benefits of COVID-19 vaccination; compared to the general population, SOT recipients remain comparatively vulnerable to COVID-19 infection, even after vaccination [1,2,3,4].

While the safety of COVID-19 vaccination has been well established for SOT recipients, its efficacy in preventing the disease or mitigating its severity in this patient group is still not fully answered [5,6,7]. Multiple studies have demonstrated low immunogenicity of the COVID-19 vaccination in SOT recipients, especially following only two doses [8,9]. Several studies have aimed to investigate morbidity and mortality outcomes of COVID-19 infection in SOT recipients following vaccination. A large United Kingdom transplant registry analysis reported a reduction of COVID-19 related mortality from 12.6% to 7.7% following two doses of COVID-19 vaccination; however, this study did not adjust other contributing variables, such as patient-specific factors or changing medical standards of care for COVID-19 [7]. An additional registry study reported an 80% reduction of symptomatic COVID-19 incidence in vaccinated SOT recipients, but was, similarly, an unadjusted analysis [10].

Along with the advent of COVID-19 vaccination, various COVID-19 infection treatment modalities have evolved over the past two years. Amongst the most popular, monoclonal antibody (Mab) therapy became available for immunocompromised groups, including SOT recipients [11]. Mab treatment has shown to decrease mortality and need for hospitalization in SOT recipients [12,13,14].

As COVID-19 treatment modalities have become increasingly used in parallel with widespread COVID-19 vaccination, it is critical to include these treatments as a variable when analyzing vaccination related outcomes. The aim of this study was to perform a single-center propensity score matching (PSM) analysis on post-COVID-19 infection survival of consecutive vaccinated and unvaccinated SOT patients at an academic transplant institution serving a highly endemic area of the United States, with low COVID-19 vaccination rates but early broad utilization of Mab treatment on eligible COVID-19 infected SOT recipients.

## 2. Methods

**Figure 1:**
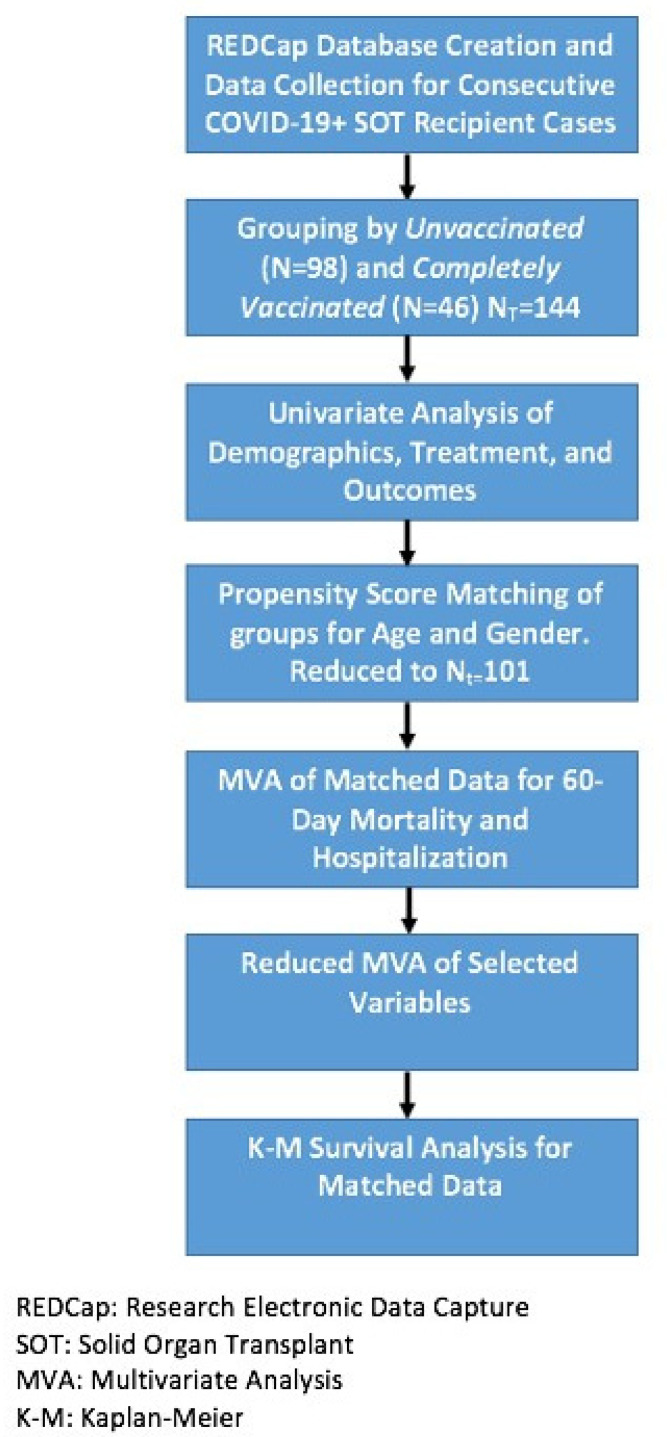
Study Flowchart.

After obtaining approval by the University of Arkansas for Medical Sciences Institutional Review Board (IRB 262269), a de-identified prospectively populated institutional database was built, registering consecutive COVID-19 positive SOT recipients from 1 February 2020 through 3 January 2022 (Study flow chart, Figure 1) [3]. Included patients were those at or above 18 years of age with a functioning liver, kidney, or simultaneous liver-kidney (SLK) allograft at the time of enrollment, who had tested positive for COVID-19 infection via nasopharyngeal polymerase chain reaction testing. Patients were entered into the database by a coded study number. Data captured included recipient demographics, comorbidities (diabetes, hypertension, obesity, coronary artery disease, cerebrovascular disease, and cancer), immunosuppression regimen, COVID-19-related hospitalization and treatment modalities, COVID-19 vaccination status, vaccination type and timing, and 60-day mortality. Patients were categorized as vaccinated vs. unvaccinated, based on their COVID-19 vaccination completion status. Complete vaccination was defined as greater than or equal to two weeks following two doses of mRNA (Pfizer or Moderna) COVID-19 vaccination or greater than or equal to two weeks following one dose of the Johnson & Johnson’s Janssen COVID-19 vaccination. Patients who had received a single dose of mRNA vaccine were categorized as unvaccinated.

Baseline characteristics between vaccinated and unvaccinated groups were analyzed. PSM analysis on age and sex was completed for vaccination status [15]. Propensity scores were estimated by logistic regression with covariates for age and sex. The matching process followed a 2:1 nearest-neighbor approach with replacement. A total of 43 unvaccinated patients were discarded before the multivariable analysis was completed. After this matching process, the standardized mean differences of the covariates were less than 0.1, which indicated an improvement in balance. The matched dataset was used to build a multivariable model with all collected variables, for which odds ratios were reported. Only significant variables were selected from this multivariate analysis to create a reduced model. Mab treatment was also included due to its clinical relevance. Reduced models were built for 60-day mortality and hospitalization. A sensitivity analysis for 60-day mortality and hospitalization was also performed for patients who had not received Mab therapy. A Kaplan–Meier (K–M) survival curve and life table were constructed from the matched dataset for a 60-day period post-COVID-19 infection diagnosis [16]. Age was checked for relative normality by looking at a histogram presenting its distribution, mean, and standard deviation. The other variables were categorical and therefore presented as frequency and percentages. Study data were collected and managed using REDCap electronic data capture tools hosted at UAMS Medical Center. Statistical analysis was performed using R Statistical Software (version 4.1.0; R core Team 2021, Vienna, Austria).

## 3. Results

N_T_ = 144 patients were eligible for enrollment, with N = 98 (68.06%) unvaccinated and N = 46 (31.94%) vaccinated. A total of 98 (86.06%) patients were unvaccinated at the time of infection. Age and sex distribution were similar among the two groups (Table 1). The majority (76.4%) of the patients had received a kidney transplant; twenty-nine patients (20.14%) had received a liver transplant; five patients (3.5%) had been SLK recipients. The distribution of organ transplant type among the two groups had been comparable (Table 1, *p* = 0.805). The distribution of the comorbidities studied (cancer, diabetes mellitus, hypertension, obesity, and coronary artery and cardiovascular disease) were similar between the two groups (Table 1). Administration of Mab was more common (58.7%) in the vaccinated group (Table 1, *p* < 0.001). The overall 60-day mortality rate was 7.6% (N = 11); 60-day mortality in the unvaccinated group was 11.2% (N = 10), compared to 2.2% (N = 1) in the vaccinated group. In unmatched data, each group had statistically similar mean age, female sex, rates of comorbid conditions, treatment with Remdesivir, hospitalization rates, and 60-day mortality. 18.4% (N = 18) of the unvaccinated received Mab treatment, contrary to 58.7% (N = 27) in the vaccinated group (*p* < 0.001) (Table 1).

PSM reduced the study population to N_T_ = 101, with N = 55 in the unvaccinated group and N = 46 in the vaccinated group. A multivariable logistic regression using the matched data for 60-day mortality showed vaccinated status (OR 0.18; *p* = 0.05), age (OR 1.20; *p* < 0.001), female sex (OR 0.16, *p* = 0.007), and kidney transplant status (OR 21.27, *p* = 0.006) to be significant prognosticators. Mab treatment (OR 0.19; *p* = 0.074) and co-morbid conditions were not significant in the 60-day mortality multivariable analysis. The reduced multivariable analysis for 60-day mortality showed age (OR 1.22; *p* < 0.001), female sex (OR 0.13; *p* = 0.005), Mab treatment (OR 0.06; *p* = 0.014), and kidney transplant status (OR 35.08; *p* < 0.001) as significant to odds of 60-day mortality (Table 2). The reduced multivariable analysis for hospitalization showed age (OR 1.05; *p* = 0.018) and kidney transplant status (OR 5.65; *p* = 0.036) to be significant hospitalization risk factors (Table 2).

Sensitivity analysis on patients who had not received Mab therapy decreased the population to N = 64 patients, with 14% mortality (N = 9) and 35.9% (N = 23) hospitalization rates. Vaccination status did not impact 60-day mortality [odds ratio (OR) 0.59, *p* = 0.59] or hospitalization rates (OR 0.83, *p* = 0.784) in this subgroup. As in the entire cohort, age (OR 1.23, *p* = 0.001) and kidney transplant status (OR 50.57, *p* = 0.003) remained significant 60-day mortality risk factors (Table 3).

**Figure 2:**
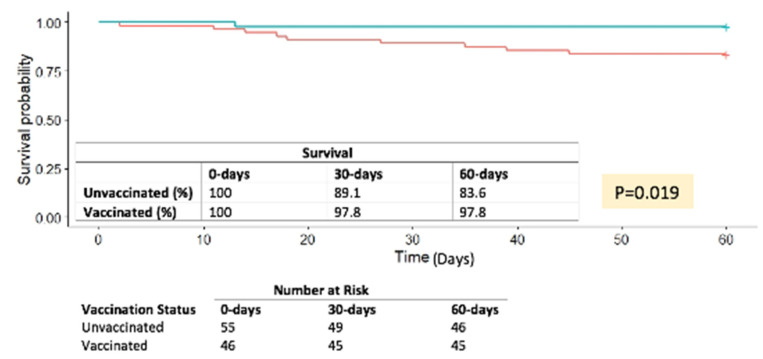
Matched Data Kaplan–Meier Survival Curve.

Matched-dataset K–M survival analysis showed an 8.7% and a 14.2% survival benefit for the vaccinated group at 30 and 60 days post-COVID-19 diagnosis, respectively (log rank *p* = 0.019) (Figure 2).

## 4. Discussion

On PSM multivariable analysis, the OR for mortality was five-fold for the unvaccinated; however, the observation did not reach significance (95%CI 0.3–1.20; *p* = 0.077). This is at least partially due to the insufficient cohort sample within the study time frame, which limited the proportion of vaccinated patients who had received more than two vaccines, since booster vaccination was not broadly available during the early stages of the registry development. Based on our data, it was unclear whether this decreased mortality trend observed in the vaccinated group is solely to be ascribed to vaccination or is in tandem with other innovative treatments, and/or the medical community becoming more adept in tackling such cases.

Mab treatment decreased mortality odds 20-fold. Notably, Mab treatment rate was three times higher among the vaccinated (58.7 vs. 18.37%). The perhaps paradoxical observation of more patients receiving Mab treatment in the vaccinated group rather the unvaccinated group has at least two explanations: Firstly, treatment selection bias. Administration of Mab on eligible transplant patients was encouraged as soon as it became available and was closely monitored by this study group and infectious disease. In our center, implementation of Mab treatment protocols almost coincided chronologically with the introduction of vaccine. As per our institutional protocol, Mab was recommended on all transplant patients presenting with mild–moderate COVID-19 and was administered at an outpatient setting. Based on that protocol, patients requiring hospitalization/presenting with severe COVID-19 would no longer qualify for Mab treatment. Since severely ill patients and those requiring hospitalization would no longer qualify for Mab treatment, it would be inescapable to observe less Mab recipients among those diagnosed with severe COVID-19; given that unvaccinated status is indeed associated with increased disease severity and need for hospitalization, Mab treatment after COVID-19 diagnosis was expected to be less likely among the unvaccinated transplant patients. Secondly, given that the percentage of transplant patients willing to undergo vaccination increased at the latter stages of the cohort, it would be more likely for a transplant patient presenting with COVID-19 to belong among the vaccinated.

This study mirrored factors known to increase odds of COVID-19 mortality. Increasing patient age has consistently shown to increase COVID-19 mortality risk [3,17,18]. Kidney transplant status (as opposed to liver transplant status) had a 40-fold odds ratio for 60-day mortality following COVID-19 infection (*p* < 0.001). Kidney transplant recipients were already known to have greater COVID-19 mortality risk compared to liver transplant recipients [4,19,20]. This seemingly protective effect of liver transplant status may be attributable to differences in immunosuppression regimen, such as the early discontinuation of antimetabolites after liver transplant, while continuing on favorable immunomodulating tacrolimus monotherapy (data not shown) or to metabolic and cardiovascular comorbidities more prevalent among the renal failure population [3,17,21].

The effect of age, female sex, and kidney transplant status on COVID-19 mortality were exemplified at the Mab sensitivity analysis, which included transplant patients infected with COVID-19 who had not received Mab treatment (Table 3). Vaccination did not stand out as a significant factor for 60-day mortality or hospitalization on sensitivity analysis, perhaps echoing previous reports of reduced reactogenicity of COVID-19 vaccination following the administration of two or less doses of mRNA vaccines on SOT patients [6,9,22].

A similar PSM study on the effect of vaccination status on SOT patients who have contracted COVID-19 was published by Hall et al., encompassing multiple transplant centers in Canada [23]. Results showed no difference in disease severity—including mortality—between vaccinated and unvaccinated [23]. Even though the Canadian study was conducted over a similar time frame, vaccinated group inclusion criteria differed: vaccinated were considered individuals who had either received one or two doses of COVID-19, even if the administered vaccine was intended as a two-dose series. Secondly, Mab treatment was not part of the Canadian PSM analysis nor seemed to be a standardized Mab treatment component on eligible patients (i.e., high risk patients presenting within a few days history of mild disease), as it has been the case at our institution. Lastly, and perhaps more importantly, since both analyses were based on registries relying on patient diagnosis of the disease in order to be included in the study, the number of COVID-19-infected patients that had asymptomatic or mild disease which was never detected and, therefore, never captured by the registries, remains unknown and possibly under-represented; it is thus conceivable that the discrepancy of apparent protective effect of vaccination among the two PSM analyses to be, at least to a certain degree, attributed to differing proportions of representation of vaccinated COVID-19 patients. Such missed data would most likely correspond to patients with mild or even asymptomatic disease—who never needed to seek medical attention and therefore be captured by the registries—and therefore better prognosis; given that these patients would be more likely to be vaccinated than not, this missing data bias would skew the outcomes in favor of the unvaccinated status. Further differences may be attributable to the inclusion of heart and lung transplant recipients by the Canadian group, among other unclear reasons [23].

Even though prior high-quality studies support that immune response improves with vaccine boosters [22,24,25], this present study did not account for differences in number of vaccination doses received by recipients beyond two (or beyond one for the Janssen vaccine). The reason is that such sensitivity analysis would have been severely impaired by the small sample size. Nonetheless, almost half of the vaccinated group (47.8%) had received their third vaccination at least two weeks prior to the time of COVID-19 diagnosis. That third vaccination may account for the survival benefit signal detected at our single-center PSM study, contrary to the multi-center study published by the Canadian group [23].

As already mentioned, the greatest limitation of this analysis has been its small sample size capturing data during a period where available treatments and knowledge over the responsiveness of the transplant population to available vaccines was still evolving. A further limitation has been the limited capturing of those asymptomatic or peripatetic SOT recipients who contracted the disease yet had never been diagnosed; as a result, the population base of the vaccinated group with mild or asymptomatic disease and, therefore, better prognosis may have been under-represented, skewing the outcomes. The number of vaccinated patients who had received a booster who, as per the latest date, would be more likely to be more effectively protected [22], was limited by the study time frame, risking blunting of the protective effect of full vaccination on this PSM analysis. Lastly, changes in COVID-19 variant throughout this study may also contribute to changes in mortality rate at various times. Alpha, Beta, Gamma, Delta, and Omicron were all possible variants throughout the course of this study, each with differing infectivity and mortality rates [26]. Patients in this study were not routinely tested for specific variants.

The study’s major strength was that data were matched for their effect on mortality and hospitalization. This study was conducted at an academic institution serving as the solitary transplant provider within a state ranked among the highest in the number of COVID-19 cases per 100,000 population for most of the pandemic, while also ranking among the lowest in vaccination rates, fifth highest for population being on dialysis, and fourth on crude mortality rates in the United States [27,28,29,30].

## 5. Conclusions

Although limited by sample size, this study showed that increased age and kidney transplant status were the most significant mortality risk factors following COVID-19 infection on SOT recipients. Vice versa, the liver transplant status, the female sex, and the administration of Mab treatment were protective. Death risk was five-fold among the unvaccinated, even though this cohort was probably not sufficiently powered to showcase this effect; plus, subject recruitment concluded prematurely perhaps compromising the demonstration of the more robust booster vaccination effect. Finally, the study displayed adjuvant Mab treatment as an emerging independent protective factor; this is a potentially significant observation, particularly with regard to the care of these highly vulnerable patients in areas with low vaccination rates.

As it has been mentioned at the study limitations, this is a retrospective, historical study providing a snapshot of the status at an earlier stage of the pandemic. Since then, treatment arrows in our quiver and our collective experience in tackling the disease have mounted, perhaps in part compensating for the expected inferior outcomes among the unvaccinated and the unsatisfactory outcomes among those partially vaccinated against COVID-19. An up-to-date comprehensive PSM study including more patients who have received booster vaccinations, as well as subjects with asymptomatic or mild breakthrough infection identified via transplant population screening would be necessary in order to provide more robust evidence of the protective effect of full COVID-19 vaccination on SOT recipients.

## Figures and Tables

**Table 1:** Demographics, Treatments, and Outcomes by Vaccine Status (N = 144).

	Unvaccinated (N (%))	Vaccinated (N (%))	*p*
**N**	98 (68.06)	46 (31.94)	
**Age (mean (SD))**	51.96 (13.53)	49.48 (12.66)	0.297
**Gender:**			
Male	59 (60.20)	23 (50.00)	0.331
Female	39 (39.80)	23 (50.00)	
**Type of Transplant:**			
Liver	21 (21.43)	8 (17.39)	0.805
Kidney	74 (75.51)	36 (78.26)	
SLK	3 (3.06)	2 (4.35)	
**Comorbidities:**			
Cancer	3 (3.06)	4 (8.70)	0.294
DM, HTN, Obesity *	82 (83.67)	4 (86.96)	0.793
CAD, CVA *	12 (12.24)	4 (8.70)	0.728
**Mab Treatment**	18 (18.37)	27 (58.70)	<0.001
**60-day mortality**	11 (11.22)	1 (2.17)	0.131

SLK: Simultaneous Liver Kidney Transplant, DM: Diabetes Mellitus, HTN: Hypertension, CAD: Coronary artery disease, CVA: Cerebrovascular event, Mab: Monoclonal antibody, * Comorbidities not mutually exclusive.

**Table 2:** Matched Data Reduced multivariable analysis for 60-day mortality and hospitalization. (a) Matched Data Reduced MVA for 60-Day Mortality (NT = 101) (b) Matched Data Reduced MVA for Hospitalization (NT = 101).

(a)			
	Odds Ratio	95% Confidence Interval	*p*
**Vaccinated**	0.19	0.03–1.20	0.077
**Age**	1.22	1.11–1.33	<0.001
**Female Sex**	0.13	0.03–0.53	0.005
Kidney Transplant *	40.93	4.53–370.12	0.001
**Mab Treatment**	0.06	0.01–0.57	0.014
**(b)**			
**Vaccinated**	1.21	0.43–3.39	0.716
**Age**	1.05	1.01–1.10	0.018
**Female Sex**	0.55	0.21–1.45	0.223
Kidney Transplant *	5.65	1.12–28.28	0.036
**Mab Treatment**	0.62	0.20–1.86	0.385

MVA: Multi-variable analysis, Mab: Monoclonal antibody, * As compared to Liver Transplants.

**Table 3:** Mab Sensitivity MVA for 60-day mortality (N = 64). (a) Mab Sensitivity Reduced MVA for 60-day Mortality (b) Mab Sensitivity Reduced MVA for Hospitalization.

(a)			
	Odds Ratio	95% Confidence Interval	*p*
**Vaccinated**	0.59	0.09–4.04	0.590
**Age**	1.23	1.09–1.38	0.001
**Female Gender**	0.12	0.02–0.67	0.017
Kidney Transplant *	50.57	3.92–651.83	0.003
**(b)**			
**Vaccinated**	0.83	0.22–3.12	0.784
**Age**	1.06	1.00–1.13	0.048
**Female Gender**	0.60	0.17–2.13	0.423
Kidney Transplant *	5.4	0.79–37.17	0.085

MVA: Multi-variable analysis, Mab: Monoclonal antibody. * As compared to Liver Transplants

## Data Availability

The data presented in this study are available on request from the corresponding author.

## Abbreviations

COVID-19	Coronavirus Disease 2019
Mab	monoclonal antibody
OR	odds ratio
PSM	propensity score matching analysis
SLK	simultaneous liver kidney transplant
SOT	solid organ transplant
